# Grape Seed Extract Attenuates Hepatitis C Virus Replication and Virus-Induced Inflammation

**DOI:** 10.3389/fphar.2016.00490

**Published:** 2016-12-21

**Authors:** Wei-Chun Chen, Chin-Kai Tseng, Bing-Hung Chen, Chun-Kuang Lin, Jin-Ching Lee

**Affiliations:** ^1^Graduate Institute of Medicine, College of Medicine, Kaohsiung Medical UniversityKaohsiung, Taiwan; ^2^Institute of Basic Medical Sciences, College of Medicine, National Cheng Kung UniversityTainan, Taiwan; ^3^Center of Infectious Disease and Signaling Research, College of Medicine, National Cheng Kung UniversityTainan, Taiwan; ^4^Department of Biotechnology, College of Life Science, Kaohsiung Medical UniversityKaohsiung, Taiwan; ^5^Institute of Biomedical Science, National Sun Yat-Sen UniversityKaohsiung, Taiwan; ^6^Doctoral Degree Program in Marine Biotechnology, College of Marine Sciences, National Sun Yat-Sen UniversityKaohsiung, Taiwan; ^7^Graduate Institute of Natural Products, College of Pharmacy, Kaohsiung Medical UniversityKaohsiung, Taiwan; ^8^Research Center for Natural Products and Drug Development, Kaohsiung Medical UniversityKaohsiung, Taiwan

**Keywords:** hepatitis C virus, grape seed extract, cyclooxygenase-2, viral replication, inflammation

## Abstract

Hepatitis C virus (HCV) infection is a causative factor leading to hepatocellular carcinoma due to chronic inflammation and cirrhosis. The aim of the study was first to explore the effects of grape seed extract (GSE) in HCV replication, and then to study mechanisms. The results indicated that a GSE treatment showed significant anti-HCV activity and suppressed HCV-elevated cyclooxygenase-2 (COX-2) expression. In contrast, exogenous COX-2 expression gradually attenuated antiviral effects of GSE, suggesting that GSE inhibited HCV replication by suppressing an aberrant COX-2 expression caused by HCV, which was correlated with the inactivation of IKK-regulated NF-κB and MAPK/ERK/JNK signaling pathways. In addition, GSE also attenuated HCV-induced inflammatory cytokine gene expression. Notably, a combined administration of GSE with interferon or other FDA-approved antiviral drugs revealed a synergistic anti-HCV effect. Collectively, these findings demonstrate the possibility of developing GSE as a dietary supplement to treat patients with a chronic HCV infection.

## Introduction

Chronic hepatitis C caused by hepatitis C virus (HCV) infection is strongly associated with the progression of liver disease, including cirrhosis and hepatocellular carcinoma (HCC), by inducing chronic liver inflammation (Kwon et al., 2014). Till date, more than 170 million people are chronically infected with HCV. HCV is an enveloped, positive-sense single-stranded RNA virus belonging to the *Flaviviridae* family. HCV genomic RNA encodes a polyprotein that is then cleaved by both host and virus protease into 10 mature proteins (core, glycoprotein E1, and E2) and non-structural proteins (NS2, NS3, NS6A, NS4B, NS5A, and NS5B). Among these proteins, core and NS5A have been suggested as potentially oncogenic proteins contributing to the development of HCC during chronic HCV infection because of long-term activation of various pro-inflammatory cytokines and chemokines, such as tumor necrosis factor (TNF-α), interleukin-1 (IL-1), inducible nitric oxide synthase (iNOS), and cyclooxygenase-2 (COX-2) (Banerjee et al., 2010). To date, there is no vaccine available to prevent HCV infection. Until recently, three direct-acting antiviral (DAA) agents targeting HCV protease or polymerase, including telaprevir, boceprevir, and sofosbuvir, have already been approved to treat HCV infection alone or in combination with current standard-of-care therapy using pegylated interferon-alfa plus ribavirin (Koretz, 2014). Although, the sustained virologic response (SVR) rate is improved with the use of these agents, the side effects profile, DAA-resistance mutations, and even the high cost frequently interfere with their therapeutic effect (Sarrazin and Zeuzem, 2010). Thus, efforts to screen molecules that focus on new therapeutic targets are still required.

Cyclooxygenase-2 is an important pro-inflammatory mediator that responses to diverse inflammatory stimuli such as a 12-*O*-tetradecanoylphorbol-13-acetate (TPA) treatment or a virus infection (Kim et al., 2010; Radi et al., 2010; Chen et al., 2015). The prostaglandins converted from arachidonic acid by COX-2 have been reported to enhance tumor growth and angiogenesis in various tumors (Chang et al., 2004; Tveteraas et al., 2012). The aberrant expression of COX-2 observed in chronic hepatitis C patients was associated with an increased risk of HCC (Bae et al., 2001). As mentioned earlier, the HCV protein greatly stimulated COX-2 expression and in turn, COX-2 overexpression enhanced HCV replication (Chen et al., 2015). Because of these observations, our previous studies have proved that several natural products can effectively suppress HCV replication by inhibiting NF-kB-mediated COX-2 expression, which supported targeting the COX-2 signaling pathway as a promising approach to develop therapeutic or chemopreventive agents against HCV-positive HCC formation (Lee et al., 2011; Lin et al., 2013).

Grape seed extract (GSE) has been widely used as dietary supplement because of its many bioactivity properties, including antioxidant, hepatoprotective, neuroprotective, cardioprotective, anticancer, anti-inflammation, antiaging, and antimicrobial effects (Bagchi et al., 2014; Olaku et al., 2015). GSE contains large amount of phenolic compounds, including gallic acid, (+)-catechin, epicatechin, dimeric procyanidin, and proanthocyanidins that are suggested to be the major bioactive components against many diseases (Shi et al., 2003). GSE has also been reported to exhibit an antiviral activity against human immunodeficiency virus type 1 (Nair et al., 2002), human enteric virus, human norovirus surrogates [feline calicivirus (FCV) F9 and murine norovirus (MNV-19)] (Su and D’Souza, 2011), and hepatitis A virus (Joshi et al., 2015), although the antiviral action was different and also not sufficiently studied. To date, its effect on HCV is undefined. Here, we assess the biological effect and molecular action of GSE on anti-HCV replication and anti-HCV-induced inflammation. Notably, the effect of combined treatment of GSE and the anti-HCV drugs currently used in a clinical setting were further assessed, which provides great clinical and economic significance for the supplemental treatment for HCV infection and its related diseases.

## Materials and Methods

### Cell Culture and Reagents

Huh-7 cells, the human hepatoma cell, and Ava5 cell, the Huh-7 cells harboring HCV genotype 1b subgenomic RNA replicon (Blight et al., 2000), were used for antiviral studies. Both were maintained in Dulbecco’s modified Eagle’s medium with 10% heat-inactivated fetal bovine serum, 1% antibiotic-antimycotic, and 1% non-essential amino acids. All cells were incubated at 37°C with a 5% CO_2_ supplement. The IH636 GSE was purchased from InterHealth Nutraceuticals (Benicia, CA, USA) (Shi et al., 2003). Interferon α-2a (Roferon-A) was purchased from Roche, Ltd. Telaprevir was purchased from Legend Stat International, Co., Ltd, and daclatasvir and sofosbuvir were purchased from Shanghai Haoyuan Chemexpress, Co., Ltd. The anti-HCV agents were prepared as stock solution at 100 mM in 100% DMSO. The final concentration of DMSO in the all experiments was constantly maintained at 0.1%. All chemicals were diluted by DMEM medium.

### Western Blotting Assay

The standard procedure of Western blotting was performed as described previously (Lee et al., 2011). The membranes were probed with monoclonal antibodies specific for HCV NS5B (1:5000; Abcam, Cambridge, MA, USA), glyceraldehyde-3-phosphate dehydrogenase (GAPDH) (1:10000; GeneTex, Irvine, CA, USA), anti-COX-2 antibody (1:1000; Cayman, MI, USA), anti-MAPK (phosphorylated and unphosphorylated forms of ERK1/2, p38, and JNK), anti-IKKα, anti-phospho-IKKα/β (Ser176/180), anti-NF-κB, anti-IκB-α, anti-phospho-IκB-α (Ser32) (1:1000; Cell Signaling Technology, Inc., Danvers, MA, USA), or anti-C-Myc antibody (1:1000; GeneTex, Irvine, CA, USA). The ECL detection kit was used for the signal detection (PerkinElmer, Shelton, CT, USA).

### Quantitative Real-Time RT-PCR (qRT-PCR) Analysis

The total RNA of the cells were isolated with a total RNA miniprep purification kit (GMbiolab, Co., Ltd, Taiwan), according to the manufacturer’s instructions. The cDNA synthesis was performed by M-MLV Reverse Transcriptase (Promega, Madison, WI, USA), according to the manufacturer’s instructions. The levels of HCV NS5B, TNF-α, IL-1β, iNOS, and COX-2 RNA were detected by RT-qPCR with the following forward and reverse primer sets: NS5B (AJ238799), 5′-GGA AAC CAA GCT GCC CAT CA-3′ and 5′-CCT CCA CGG ATA GAA GTT TA-3′; TNF-α (NM_000594), 5′-CCT GTG AGG AGG ACG AAC-3′ and 5′-AAG TGG TGG TCT TGT TGC-3′; IL-1β (NM_000576), 5′-GGA GAA TGA CCT GAG CAC-3′ and 5′-GAC CAG ACA TCA CCA AGC-3′; iNOS (NM_000625), 5′-CTT TGG TGC TGT ATT TCC-3′ and 5′-TGT GAC CTC AGA TAA TGC-3′; and COX-2 (NM_000963), 5′-CCG AGG TGT ATG TAT GAG-3′ and 5′-TGG GTA AGT ATG TAG TGC-3′. The relative RNA level of these genes in each sample were normalized to cellular GAPDH mRNA with the forward primer: 5′-GTC TTC ACC ACC ATG GAG AA-3′ and reverse primer: 5′-ATG GCA TGG ACT GTG GTC AT-3′. The relative expression levels were analyzed by the ABI Step One Real-Time PCR-System in the standard procedure (ABI Warrington, UK).

### Cytotoxicity Assay

The relative cell viabilities were determined by the CellTiter 96^®^ AQueous One Solution Cell Proliferation Assay (MTS) assay that depended on the measurement of mitochondria dehydrogenase enzyme activity from viable cells (Promega Corporation, Madison, WI, USA). Ava5 cells at the density of 5 × 10^3^ per well were treated with GSE at different concentrations. After a 3-day incubation, the supernatant was removed and added to the MTS mixture with 100 μl of phenol red-free medium and 20 μl of the MTS reagent at 37°C. After 4 h, the relative cell viabilities were analyzed by measuring the absorption at 490 nm on a microplate reader.

### HCV JFH-1 Infection Assay

The cell culture-produced HCV (HCVcc) were generated by the transfection of *in vitro* transcribed full-length JFH-1 RNA into Huh-7.5 (Kato et al., 2006). The Huh-7 cells with a density of 5 × 10^4^ per well were infected with JFH-1 HCVcc at a multiplicity of infection (MOI) of 0.1 for 8 h. At the end of infection, the supernatant was removed and the cells were incubated with various concentrations of GSE for an additional 3 days. The total RNA of the cells was isolated and the relative HCV RNA levels were analyzed by a Step One Real-Time PCR-System.

### Synergy Analysis Synergistic Isobologram

Ava5 cells were incubated in a mixture containing GSE (0, 2.5, 5, 10, and 20 μg/ml) in combination with each of the anti-HCV agents, IFN-α (0, 7.5, 15, 30, and 60 U/ml), HCV NS3/4A protease inhibitor telaprevir (0, 0.075, 0.15, 0.3, and 0.6 μM), HCV NS5A inhibitor daclatasvir (0, 1, 2, 4, and 8 pM), and the RNA-dependent RNA polymerase nucleoside inhibitor sofosbuvir (0, 10, 20, 40, and 80 nM). After 3 days, the relative HCV RNA levels were analyzed by a Step One Real-Time PCR-System for calculating the drug dose effects. Based on the method of Chou and Talalay (1984), the combination index (CI) was calculated by CalcuSyn software (Biosoft, Cambridge, UK) with the presence of synergism (CI < 1), additivity (CI = 1), and antagonism (CI > 1).

### Plasmid Construction

The inactive COX-2 mutant (S516Q) expression vector (pCMV-COX-2^mut^-Myc) without cyclooxygenase activity were generated from pCMV-COX-2-Myc with the primers: forward: 5′-GTTGGAGCACCATTC*CAG*TTGAAAGGACTTATG-3′, reverse: 5′-CATAAGTCCTTTCAA*CTG*GAATGGTGCTCCAAC-3′ by QuikChange^®^ Site-Directed Mutagenesis Kit according to the manufacturer’s protocol (Stratagene, La Jolla, CA, USA) as previous described (Lecomte et al., 1994). All of the DNA fragments were confirmed by DNA sequencing.

### Transient Transfection and Luciferase Activity Assay

All the transfection reactions were performed with a T-Pro^TM^ reagent (Ji-Feng Biotechnology, Co., Ltd, Taiwan), in accordance with the manufacturer’s instructions. The Ava5 cells with a density of 5 × 10^4^ per well were transfected with pCOX-2-Luc or pNF-κB-Luc (BD Biosciences Clontech, Palo Alto, CA, USA) for 8 h. After the transfection procedure, the medium was replaced with various concentrations of GSE for 3 days. The luciferase assay was performed using the Bright-Glo^TM^ Luciferase Assay System (Promega) according to the manufacturer’s protocol with cell extracts from each sample. To identify COX-2 regulation by GSE, Ava5 cells were transfected with indicated concentrations of the COX-2 expression vector (pCMV-COX-2-Myc) from 0.5 to 2 μg, and then treated with GSE for additional 3 days. To evaluate the effect of GSE on viral-induced inflammation response, the parental Huh-7 cells were infected with the JFH-1 virus for 8 h or transfected with HCV core or NS5A expression vector (pCMV-Core-Myc; pCMV-NS5A-Myc) for 12 h, followed by incubation with or without GSE (5 and 20 μg/ml) for another 3 days. To determine the effect of GSE on virus-induced inflammatory genes (TNF-α, IL-1β, iNOS, and COX-2), Huh7 cells were infected with JFH-1 or transfected with pCMV-Core-Myc or pCMV-NS5A-Myc in the presence of GSE for 3 days. Subsequently, the total RNA of the cells was isolated and the relative inflammation cytokine RNA levels were analyzed by a Step One Real-Time PCR-System.

### Intracellular Prostaglandin E2 (PGE_2_) Measurements

Ava5 or Huh7 cells were treated with GSE at various concentrations for 3 days. Subsequently, the treated cells were washed thoroughly with cold phosphate-buffered saline (pH 7.4), and intracellular PGE_2_ was extracted by a lysis reagent containing C_15_H_34_BrN to rupture cell membrane. The intracellular PGE_2_ level was measured with the PGE_2_ enzyme-linked immunosorbent assay system (Biotrak, Amersham Bioscience), according to the manufacturers protocol.

### Preparation of Nuclear Fraction

Nuclear extracts were prepared using hypotonic [10 mM HEPES, 1.5 mM MgCl_2_, 10 mM KCl, 0.5 mM DTT, 10% Nonidet P-40 (pH 7.9)] and high-salt buffer [20 mM HEPES, 1.5 mM MgCl_2_, 0.2 mM EDTA, 0.6 M KCl, 0.5 mM DTT (pH 7.9)] extraction as previously described (Abmayr et al., 2006). Briefly, Ava5 cells with a density of 4 × 10^5^ per well were treated with or without GSE at the indicated dose. After 3 days of incubation, the cells were lysed using the ice-cold hypotonic buffer. The cytoplasmic fraction were separated by centrifugation at 7,000 × *g* for 15 min. Then, the resulting nuclear pellets were extracted with high-salt nuclear extraction buffer at 4°C for 30 min and the nuclear proteins were collected after centrifugation at 20,000 × *g* for 15 min, which stored at -80°C until use. The protease inhibitors were added to hypotonic buffer and high-salt buffer immediately before use.

### Statistical Analysis

The results are presented as the mean ± SD for of least three independent experiments. Statistical data comparisons were analyzed with the Student’s *t*-test and ANOVA by using GraphPad software and *^∗^P* < 0.05 or *^∗∗^P* < 0.01 was considered statistically significant.

## Results

### GSE Suppresses HCV Replication

**FIGURE 1:**
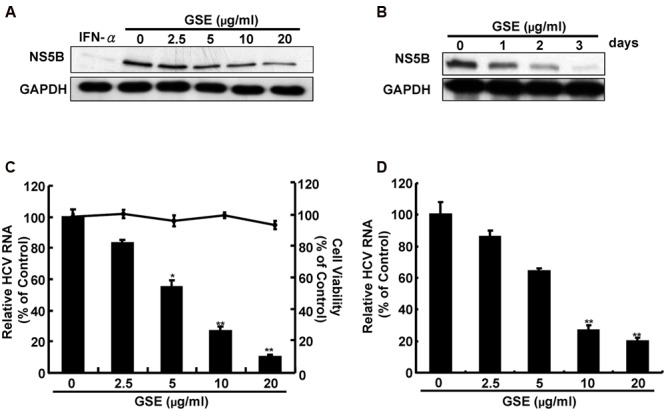
**Grape seed extract (GSE) suppresses hepatitis C virus (HCV) protein synthesis and RNA replication. (A,B)** HCV protein expression was reduced by a GSE treatment in the HCV replicon system. Ava5 cells were treated with GSE at increasing concentrations (0–20 μg/ml) for 3 days **(A)** or incubated with a different time course (1–3 days) with 20 μg/ml of GSE **(B)**. A 100 U/ml treatment of IFN-α served as the positive control for anti-HCV activity. HCV protein synthesis was detected by Western blotting with anti-HCV NS5B and anti-GAPDH antibodies. The GAPDH protein levels showed equal loading of cell lysates. HCV RNA replication was reduced by a GSE treatment in the HCV **(C)** replicon and **(D)** infectious systems. The total RNA of GSE-treated Ava5 cells or JFH-1-infected cells were extracted and the relative HCV RNA levels were quantified by qRT-PCR. The cellular *gapdh* mRNA was used as an internal control for the equal loading of cDNA. Cytotoxicity was detected by the MTS assay, as described in Section “Materials and Methods.” The relative HCV RNA level and cell viability were presented as percentage changes compared to the GSE-untreated cells which represented 100%. Data are represented as the mean ± SD for three independent experiments. ^∗^*P* < 0.05; ^∗∗^*P* < 0.01.

We first examined the anti-HCV activity of GSE using the HCV subgenomic RNA stably expressing cell line (Ava5). To this end, Ava5 cells were treated with various concentrations of GSE for 3 days or 20 μg/ml of GSE for 1–3 days, followed by Western blotting and qRT-PCR. The cytotoxicity of GSE was determined using an MTS assay under identical conditions. We found that GSE significantly inhibited HCV protein synthesis (**Figures 1A,B**) and RNA replication (**Figure 1C**) in a concentration- and time-dependent manner, and no significant cytotoxicity was observed. The inhibitory effect of GSE on HCV replication was also consistently observed in HCV-infected Huh-7 cells, with an IC_50_ of 7.5 ± 0.3 μg/ml (**Figure 1D**).

### GSE Suppresses HCV Replication by Reducing COX-2 Expression

**FIGURE 2:**
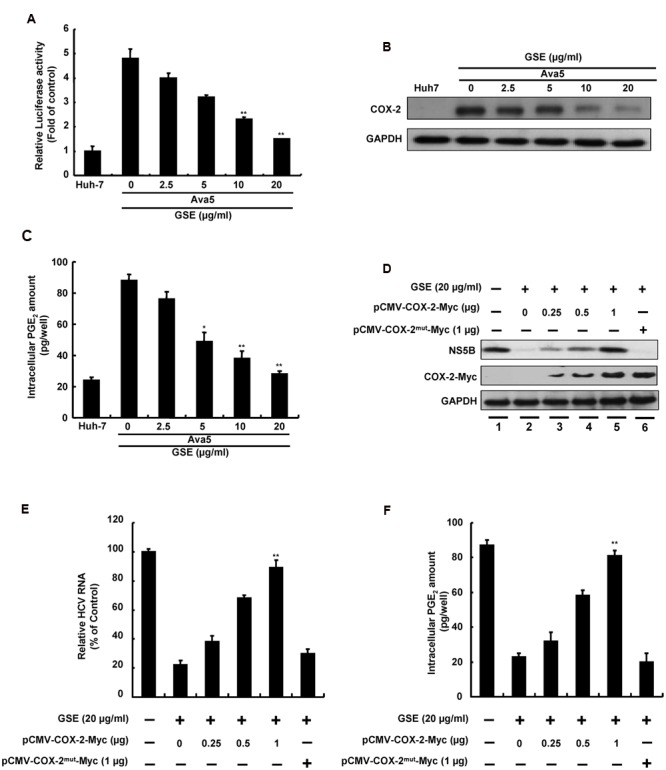
**Reduction of COX-2 expression is involved in the inhibitory effect of GSE on HCV replication. (A)** COX-2 promoter activity was reduced with a GSE treatment in a concentration-dependent manner. Ava5 cells were transiently transfected with the reporter vector containing COX-2 promoter, pCOX-2-Luc. After 8 h transfection, the medium was replaced with indicated concentrations (0–20 μg/ml) of GSE for another 3 days. Subsequently, the extracted lysates of transfected cells were analyzed by a luciferase assay. The relative COX-2 promoter activity was presented as fold changes compared to parental Huh-7 cells, whose activity was presented as 1. **(B)** Ava5 cells were treated with GSE at increasing concentrations for 3 days. The extracted cell lysates were used to analyze protein expression by Western blotting with anti-COX-2 and anti-GAPDH (loading control) antibodies, respectively. **(C)** Ava5 cells were treated with an increasing GSE concentration. After 3 days, the intercellular PGE_2_ was extracted by the lysis buffer and quantified by the PGE_2_ enzyme immunoassay system. **(D–F)** Exogenous COX-2 overexpression attenuated the anti-HCV activity of GSE in Ava5 cells. Ava5 cells were transfected with indicated amounts of pCMV-COX-2-Myc or pCMV-COX-2^mut^-Myc for 8 h. Subsequently, the transfected cells were treated with 20 μg/ml GSE for another 3 days. **(D)** The extracted cell lysates were used to analyze the protein expressions by Western blotting with anti-NS5B, anti-Myc, and anti-GAPDH (loading control) antibodies. **(E)** The extracted total RNA was used to quantified HCV RNA levels following normalization of cellular *gapdh* mRNA by qRT-PCR. The relative HCV RNA levels were presented as percentage changes compared to GSE-untreated/untransfected Ava5 cells, which was considered as 100%. **(F)** The intercellular PGE_2_ level were assayed as previous described. Data are represented as the mean ± SD for three independent experiments. ^∗^*P* < 0.05; ^∗∗^*P* < 0.01.

Previous studies have reported that GSE exhibited a protective effect against cancer development and inflammation through the inhibition of the COX-2 signaling pathway (Derry et al., 2013). Besides, more reports demonstrated that suppression of HCV-elevated COX-2 expression could effectively block HCV replication (Trujillo-Murillo et al., 2007; Lee et al., 2011). To examine whether the anti-HCV activity of GSE correlates with suppression of the COX-2 expression, we first examined the effect of GSE on COX-2 expression using COX-2 promoter-driven reporter assay in GSE-treated Ava5 cells. The results revealed that GSE gradually decreased the luciferase activity compared to the parental Huh-7 cells and the GSE-untreated Ava5 cells (**Figure 2A**). The similar result were also observed in HCV infection assay (Supplementary Figure S1A). We next examined the COX-2 protein synthesis by Western blotting and measured the intracellular amount of COX-2 metabolite PGE_2_ using the PGE_2_ ELISA assay. Consistent with the COX-2 promoter-based activity assay, GSE decreased the HCV-elevated COX-2 protein (**Figure 2B**) and PGE_2_ levels (**Figure 2C**) in a concentration-dependent manner. To investigate whether suppression of COX-2 expression contributes to the anti-HCV activity of GSE, we transfected increasing concentrations of the COX-2 expression vector pCMV-COX-2-Myc or inactive COX-2 mutant vector pCMV-COX-2^mut^-Myc into GSE-treated Ava5 cells followed by a measurement of HCV protein synthesis and RNA replication after 3 days. As shown in **Figure 2D**, an increasing overexpression of extraneous COX-2-Myc gradually restored HCV protein levels (lanes 3–5) compared to GSE-untreated (lane 1), GSE-treated cells alone (lane 2), and GSE-treated cells with extraneous inactive COX-2-Myc mutant expression (lane 6), indicating that COX-2 function is essential to support HCV replication. Similar results were obtained from the measuring the HCV RNA level under identical conditions (**Figure 2E**). We also observed that the GSE-reduced-PGE_2_ level was gradually restored by functional COX-2 expression, but not by inactive COX-2 mutant expression (**Figure 2F**). Taken together, these results revealed that GSE inhibited HCV replication by suppressing HCV-elevated COX-2 expression.

### GSE-Mediated Regulation of NF-kB and MAPK Pathways Involved in Suppressing HCV Replication

**FIGURE 3:**
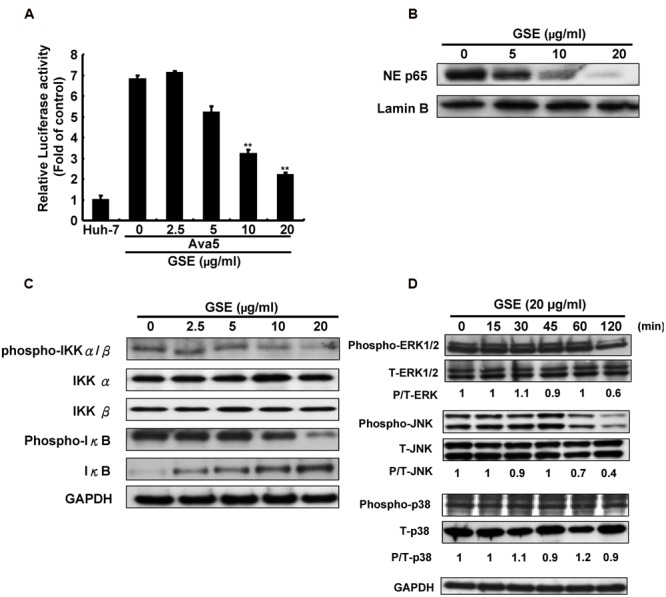
**Grape seed extract reduced NF-κB transactivity and MAPK phosphorylation for suppression of COX-2 expression in HCV replicon cells. (A)** GSE reduced NF-κB transactivity in Ava5 cells. Ava5 cells were transiently transfected with pNF-κB-Luc, which contained an NF-κB binding element linked firefly luciferase reporter gene. The pNF-κB-Luc-transfected cells were treated with 20 μg/ml of GSE for 3 days. Subsequently, the extracted lysates of transfected cells were analyzed by luciferase activity assay. The relative NF-κB transactivity was presented as fold changes compared to parental Huh-7 cells in which luciferase activity was presented as 1. The GSE treatment downregulated **(B)** NF-κB phosphorylation and **(C)** the HCV-induced NF-κB signaling pathway. Ava5 cells were treated with GSE in different concentrations (0–20 μg/ml) for 3 days and the nuclear lysates were isolated as described in Section “Materials and Methods.” The nuclear translocation of NF-κB were analyzed by Western blotting with anti-phospho-p65 and anti-Lamin B (loading control) antibodies. The effects of GSE on NF-κB regulation were analyzed by Western blotting with various antibodies against IKKα, phospho-IKKα/β, IκB-α, phospho-IκB-α, and GAPDH (loading control). **(D)** GSE treatment reduced the phosphorylation level of ERK and JNK. Ava5 cells were treated with 20 μg/ml of GSE and the lysates extracted at the indicated time points after the treatment. The protein expressions were analyzed by Western blotting with antibodies against MAPK (ERK1/2, p38, and JNK), phospho-MAPK (p-ERK1/2, p-p38, and p-JNK), and GAPDH (loading control). Data are represented as the mean ± SD for three independent experiments. ^∗^*P* < 0.05; ^∗∗^*P* < 0.01.

Both NF-κB and the mitogen-activated protein kinase (MAPK) signaling pathways are important regulators of COX-2 expression (Tsatsanis et al., 2006). To investigate whether GSE suppresses COX-2 expression by interfering with NF-κB activation, we first performed an NF-κB-mediated transcription reporter assay. The Ava5 cells were transfected a pNF-κB-Luc reporter plasmid and then incubated with increasing concentrations of GSE for 3 days. We found that the GSE concentration-dependently decreased luciferase activity compared to parental Huh-7 cells and GSE-untreated Ava5 cells (**Figure 3A**), indicating that GSE inhibited the HCV-stimulated NF-κB transcriptional activation. The similar results were also observed in HCV infection assay (Supplementary Figure S1B). A Western blot analysis further confirmed that the nuclear translocation of NF-κB p65 subunit was significantly interfered by GSE at a concentration of 20 μg/ml (**Figure 3B**). Phosphorylation of IκB by the active phosphorylated form of IKKα/β kinase is a key mechanism to control the nuclear translocation of the free form of NF-κB due to the ubiquitin-proteasome proteolysis of phospho-IκB (Chen, 2005). We further examined the effect of GSE on IKKα/β kinase and IκB phosphorylation in Ava5 cells. The results of Western blotting showed that GSE suppressed the amount of active phospho-IKKα/β kinase in a concentration-dependent manner (**Figure 3C**, upper panel). The total amount of IKKα or IKKβ did not change significantly. As expected, the phospho-IκB level was gradually decreased by GSE. In the meantime, the amount of unphospho-IκB accumulated (**Figure 3C**, middle panel). These results revealed that inactivation of the IKKα/β kinase was involved in the suppression of NF-κB-mediated COX-2 expression by a GSE treatment. In addition to the NF-κB signaling pathway, we next explored the effect of GSE on the MAPK pathways on the induction of COX-2 expression. Ava5 cells were treated with GSE at 20 μg/ml for 0–120 min and we then examined the effect of GSE on the phosphorylation status of the major regulators of the MAPK signaling pathway, including extracellular regulated protein kinases 1 and 2 (ERK1/2), p38 kinase, and c-Jun NH2-protein kinase (JNK). As shown in **Figure 3D**, GSE suppressed the amount of phosphor-ERK and phospho-JNK in a time-dependent manner (upper and middle panels). In contrast, there was no significant effect on the phospho-p38 protein level in GSE-treated Ava5 cells compared to the GSE-untreated control (lower panel). Consistent with the results of replicon system, GSE significantly suppressed the amount of phosphor-ERK and phospho-JNK instead of phospho-p38 in HCV infectious system (Supplementary Figure S2). Taken together, these results reveal that GSE inhibits HCV replication by blocking NF-κB and ERK/JNK MAPK signaling pathways to suppress COX-2 expression.

### GSE Attenuates Virus-Induced Pro-inflammation Cytokines

**FIGURE 4:**
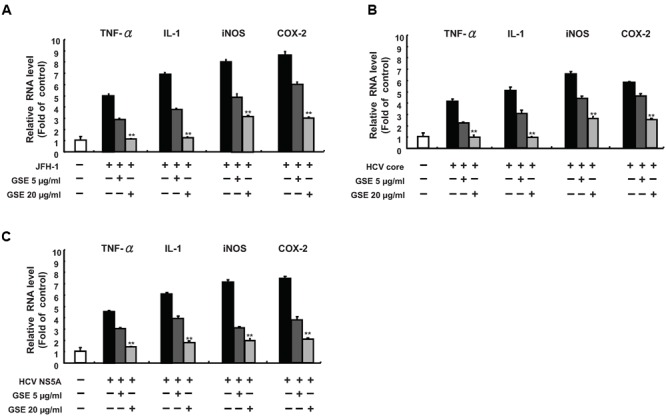
**Grape seed extract inhibits HCV viral-induced pro-inflammatory cytokine gene expression.** A GSE treatment reduced the inflammation response, which was induced by **(A)** JFH-1 infection or exogenous overexpression of **(B)** Core or **(C)** NS5A. The Huh-7 cells were infected with JFH-1 or transfected with each HCV viral protein expression vectors (pCMV-NS5A-Myc or pCMV-Core-Myc). Subsequently, the cells were treated with 5 or 20 μg/ml GSE. After 3 days, the total RNA of the cells was extracted. The inflammation cytokines including TNF-α, IL-1β, iNOS, and COX-2 were quantified with specific primers and normalized by cellular *gapdh* mRNA using qRT-PCR. The relative inflammation cytokine expression levels of uninfected/untransfected Huh-7 cell were defined as 1. Data are represented as the mean ± SD for three independent experiments. ^∗^*P* < 0.05; ^∗∗^*P* < 0.01.

Acute or chronic inflammation is one of the hallmarks to hepatic injury, viral replication, and HCC development (Sukowati et al., 2016). HCV core and NS5A protein are known to be risk factors in the development of HCV-induced chronic inflammation (Nunez et al., 2004). To examine whether GSE exhibits a hepatoprotective activity against virus-induced inflammation, we measured the effect of GSE on the mRNA levels of several inflammatory mediators, including TNF-α, iNOS, COX-2 and IL-1 in HCV-infected, HCV core-expressing, or NS5A-expressing Huh-7 cells by a qRT-PCR analysis. As shown in **Figure 4**, the results demonstrated that HCV infection (A), core (B), or the NS5A (C) protein could transcriptionally activate those of the inflammatory mediators by approximately five to eightfold as compared to parental Huh-7 cells. With a GSE treatment, the mRNA levels of those stimulated pro-inflammatory mediators were concentration-dependently reduced by GSE compared to the levels observed in HCV-infected, core or NS5A-transfected Huh-7 cells with no GSE treatment, revealing that GSE can be used as a potential hepatoprotective agent against HCV-stimulated inflammation.

### Synergistic Anti-HCV Replication by a GSE Treatment Combined with Either IFN-α or Viral Enzyme Inhibitors

To examine the potency of GSE with supplementary usage, we examined the anti-HCV activity of GSE in combination with IFN-α or other USA Food Drug Administration (FDA) approved anti-HCV drugs, including NS3/4A protease inhibitor telaprevir, NS5A inhibitor daclatasvir, and NS5B polymerase inhibitor sofosbuvir. Ava5 cells were incubated with GSE and combined with either IFN-α or other HCV inhibitors at various fixed concentration ratios. The anti-HCV replication activities were analyzed by qRT-PCR. The CI values were calculated by CalcuSyn^TM^ software based on the principle described by Chou and Talalay (Chou and Talalay, 1984). The drug interaction in terms of synergism, CI < 1 for ED50, ED75, and ED90 (range: 0.86–0.34) and shown in **Table 1**, which indicated that a combination treatment exhibited a synergistic effect on anti-HCV activity. This means that GSE could be developed as a useful supplement or adjuvant to treat patients with chronic HCV infection under a combination regimen.

**Table 1:** The CI of a GSE treatment combined with various anti-HCV agents on anti-HCV replication.

Combination compound	CI values at			Influence
	ED50	ED75	ED90	
IFN-α	0.73	0.49	0.38	Synergistic
Telapriver	0.82	0.68	0.56	Synergistic
Daclatasvir	0.86	0.72	0.53	Synergistic
Sofobuvir	0.54	0.43	0.34	Synergistic

*Ava5 cells were treated with GSE combined with IFN-α, telaprevir, daclatasvir, or sofosbuvir in a fixed ratio for 3 days. The combination treatment of anti-HCV activity was analyzed by qRT-PCR. The CI value for the effective dose of 50% (ED_*50*_), 75% (ED_*75*_), or 90% (ED_**90**_) inhibition was calculated by using the CalcuSyn computer program. Results are expressed as the mean ± SD for three independent experiments. The calculated CI values indicated that CI < 1, CI = 1, and CI > 1 represented synergism, additivity, and antagonism on anti-HCV activity between the two agents, respectively.*

## Discussion

Grape seed extract (GSE) containing more amounts of flavonoids have been promoted as a healthy food in our daily diet. It is a key issue to be resolved for establishing the bioavailability in *in vivo* conditions. In the present study, we clearly demonstrated that GSE exerted inhibitory effects on HCV replication without causing host cellular toxicity (**Figure 1**). In the last decade, there are more than 50% of plant-derived compounds have served as an important source and being used in clinical practice as pharmaceuticals for drug development (Cragg and Newman, 2005). Besides, plants are considered as potential sources to discover new bioactive compounds against HCV infection. In addition, many natural products show significant cytoprotective effects in various diseases as well as that GSE have been studied and showed significant effects in many disease, including cancer, inflammation, and even infectious diseases. Comparable with present study, there are several compounds which have significant antioxidant activity against HCV infection, such as curcumin, silymarin, silibinin, and epigallocatechin-3-gallate (EGCG) (Chen C. et al., 2012; Chen M.H. et al., 2012; Polyak et al., 2013). According to the significant antioxidant activity and popular in dietary supplement of GSE, we suggested that GSE may serve as a potential anti-viral adjuvant for HCV patients.

Grape seed extract contains a high amount of proanthocyanidins, which have been isolated from blueberry leaves and shown to inhibit HCV replication by binding to host heterogeneous nuclear ribonucleoprotein (hnRNP) A2/B1 (Takeshita et al., 2009). Although, the precise mechanism of binding hnRNPs by proanthocyanidin to suppress HCV replication was not sufficiently defined, we concluded that targeting the COX-2 signal pathway and hnRNP A2/B1 might contribute to the synergistic anti-HCV activity of GSE. Previous studies have also demonstrated that GSE exhibited the ability to inhibit azoxymethane-induced colon tumorigenesis and melanoma cell invasiveness through suppression of MAPK and NF-κB-mediated COX-2 expression (Vaid et al., 2011; Derry et al., 2013). Activation of the MAPK and NF-κB signaling pathways have been shown to be associated with the enhancement of viral replication and HCC formation (Menzel et al., 2012; Yang et al., 2015). In Trujillo-Murillo et al. (2007) study, the HCV genomic RNA and COX-2 were co-transfected in Huh-7 cell and the viral RNA replication were elevated with COX-2 induction in concentration-dependent manner. Besides, we recently showed that suppression of COX-2 could be a useful approach for development of HCV inhibitors (Chen et al., 2015; Lin et al., 2015). Furthermore, COX-2 metabolite PEGs is a risk factor to develop HCC (Loginov and Vysotskaia, 1995). Therefore, GSE could serve as a dietary supplement to prevent HCV-related carcinogenesis by targeting the COX-2 signaling pathway (**Figure 2**). Because GSE contains various bioactive constituents, we still need to investigate other ingredients against cellular targets that participate in the anti-HCV response and anti-inflammatory activities of GSE.

The viral-induced inflammation cytokines are alternative crucial risk factors for the pathophysiology of liver diseases, such as alcoholic hepatitis and HCV-related diabetes (Knobler and Schattner, 2005). In addition, the aberrent production of pathogenic pro-inflammatory cytokines and chemokines caused by HCV infection contributes to the development of HCC (Zampino et al., 2013). In this study, we found that GSE treatment significantly eliminated viral-induced cytokines production, such as COX-2, iNOS, TNF-α, and IL-1β, in either HCV protein overexpressed or JFH-1-infected Huh-7 cells (**Figure 4**). In this regard, it is notable observation against viral-induced inflammation by GSE instead of lipopolysaccharides (LPSs), ethanol or CCl_4_ -induced inflammation studies. According to the inhibition of MAPK and NF-kB in numerous anti-inflammation studies of GSE, we suggested that the reductive effect of GSE on numerous HCV-induced pro-inflammatory genes expressions may be partly mediated via NF-κB inhibition (**Figure 3**). In addition, there are also demonstrated that antioxidant therapy may have cytoprotective effects against acute or chronic inflammation and beneficial effects against viral hepatitis. Previous studies demonstrated the remarkable hepatoprotective effects of GSE in offering an enhanced antioxidant response associated with the regulation of cell proliferation, apoptosis, lipogenesis, fatty acids oxidation, and diabetes in liver disease (Ray et al., 1999; Belviranli et al., 2015; Madi Almajwal and Farouk Elsadek, 2015; Bak et al., 2016). Therefore, it is still needed to identify whether GSE-induced antioxidant response participating in the anti-HCV response and anti-inflammatory activities of GSE because the compounds may possibly contain various active constitutes. However, GSE could be administered as a hepatoprotective supplement in combination with anti-HCV-induced inflammatory drugs to prevent HCV-related diseases.

In the current therapy, problem still exist in the widely used IFN-based therapy, including an unsatisfactory curing rate and severe side effects. Very recently, an IFN-free therapy with a viral target’s drug also presents the possibility of drug-resistance, which will be a major challenge of its therapeutic effectiveness (Peter and Nelson, 2015). A combination treatment based on different targets against viral and host genes will possibly reduce the side effects and drug resistance, and even the high therapeutic cost. Besides, the host targets were considered as promising anti-viral therapy in the future. Miravirsen, an inhibitor targeting mir-122 which interacted with HCV 5′ UTR, is completed the enrollment in phase 2 clinical trail (Ottosen et al., 2015). In addition, instead of viral gene products, there are more benefits to higher barrier to viral resistance and broader genotype specificity by targeting host cell factors with specific anti-viral action. Targeting the host factor by GSE is beneficial to avoid drug resistance and to sustain the viral response because the mutation rate of the host genome is lower than that of the RNA virus genome. In addition, targeting host cellular factors required for six types of HCV replication will overcome HCV quasispecies over long-term regimens. Here, we reported that GSE can be used in combination with anti-viral inhibitors that providing synergistic anti-HCV effects (**Table 1**), and the results revealed that it is worthwhile to further investigate the effects of GSE on preventing the escape of mutant variants, different genotypes, and alleviating the side effects.

**FIGURE 5:**
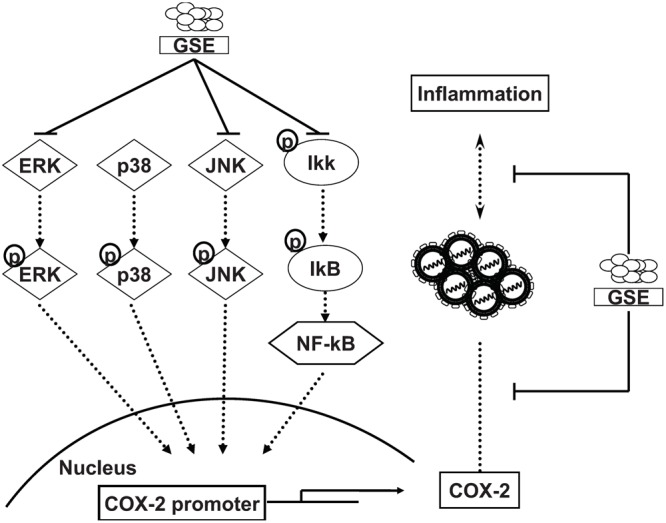
**Proposed model illustrating the mechanisms of inhibition activity of GSE on HCV replication and HCV-induced inflammation response.** GSE suppresses HCV replication by down-regulating the COX-2 expression through reducing the NF-κB and MAPK/ERK/JNK signaling pathways. Moreover, GSE significantly reduced the virus-induced inflammation response upon HCV infection or oncogenic viral protein overexpression.

Grape seed extract is rich in polyphenols and contains mixtures of monomers, oligomers (also known as procyanidin), and polymers of catechin and/or epicatechin, in which several monomer catechin derivatives, including catechin, epicatechin, (-)-epigallocatechin-3-gallate (EGCG), and quercetin, exert anti-HCV activity (Chen C. et al., 2012; Khachatoorian et al., 2012; Lin et al., 2013). However, these flavonol and polyphenol monomers are poor composed in GSE (less than 8%) and showed much higher effective concentrations against HCV replication (Overman et al., 2010). Otherwise, Procyanidin B1, the main oligomers of GSE (more than 74%), has been shown to effectively suppress HCV replication (Bentivegna and Whitney, 2002; Li et al., 2010). Therefore, we suggested that procyanidins are the potential active indicators of GSE against HCV. Previous studies reported that the oligomerized grape seed polyphenols are more conductive to absorption rate than non-oligomerized forms (Fujii et al., 2007). It is worthwhile to identify the bioactive oligomerized grape seed polyphenols responsible for anti-HCV replication for the development of anti-viral therapy. Besides, the oligomerized grape seed polyphenols demonstrated significant anti-inflammation activity through reducing NF-kB transactivation and JNK/ERK activation in macrophages (Overman et al., 2010; Sakurai et al., 2010), which is consistent with our results (**Figures 3** and **4**). In the present study, we proposed the model of GSE on anti-HCV activity by suppression of cellular COX-2 expression through the inactivation of NF-κB and ERK/JNK MAPK signaling pathways (**Figure 5**). Currently, several compounds, such as erlotinib, alisporivir, and targeting host factor strategies are in the clinical development phase for therapeutic HCV infection. We believe that GSE should be considered a potential antiviral supplement that is worthy of further investigation of anti-HCV effects using suitable animal models.

## Author Contributions

WC-C performed experiments, summarized and analyzed data and wrote the manuscript. C-KT, B-HC, and C-KL performed experiments, reviewed and edited the manuscript. WC-C and J-CL designed the experiments and edited the manuscript.

## Conflict of Interest Statement

The authors declare that the research was conducted in the absence of any commercial or financial relationships that could be construed as a potential conflict of interest.
