# Supplementary material for: Grape Seed Extract Attenuates Hepatitis C Virus Replication and Virus-Induced Inflammation
Source: Front Pharmacol. 2016 Dec 21;7:490. doi: 10.3389/fphar.2016.00490 (PMC5174132; doi:10.3389/fphar.2016.00490)

Grape seed extract attenuate hepatitis C Virus replication and virus-induced inflammation

Wei-Chun Chen[1], Chin-Kai Tseng[2,3], Bing-Hung Chen[4,5], Chun-Kuang Lin[6], Jin-Ching Lee[1,4,7,8*]

***Corresponding authors:** Jin-Ching Lee

E-mail: jclee@kmu.edu.tw

Supplementary Figure S1.

COX-2 promoter activity and NF-κB transactivity were reduced with a GSE treatment upon JFH-1 infection. (A, B) Huh-7 cells were transiently transfected with pCOX-2-Luc **(A)** or pNF-κB-Luc **(B)** for 8 h. Subsequently, the transfected cells were infected with or without JFH-1. After 8 h, the medium was replaced with indicated concentrations (0–20 μg/ml) of GSE for another 3 days. Finally, the extracted lysates of transfected cells were analyzed by a luciferase assay. The relative COX-2 promoter activity was presented as fold changes compared to parental Huh-7 cells, whose activity was presented as 1. Data are represented as the mean ± SD for three independent experiments. *$P < 0.05$; **$P < 0.01$.

Supplementary Figure S2.

GSE reduced ERK and JNK phosphorylation for suppression of COX-2 expression upon JFH-1 infection.

Huh-7 cells were infected with or without JFH-1. After 3 days, the medium was replaced with 20 μg/ml of GSE and the lysates were extracted at the indicated time points after the treatment. The protein levels were analyzed by Western blotting with antibodies against MAPK (ERK1/2, p38, and JNK), phospho-MAPK (p-ERK1/2, p-p38, and p-JNK), and GAPDH (loading control).

Supplementary Figure S1

A

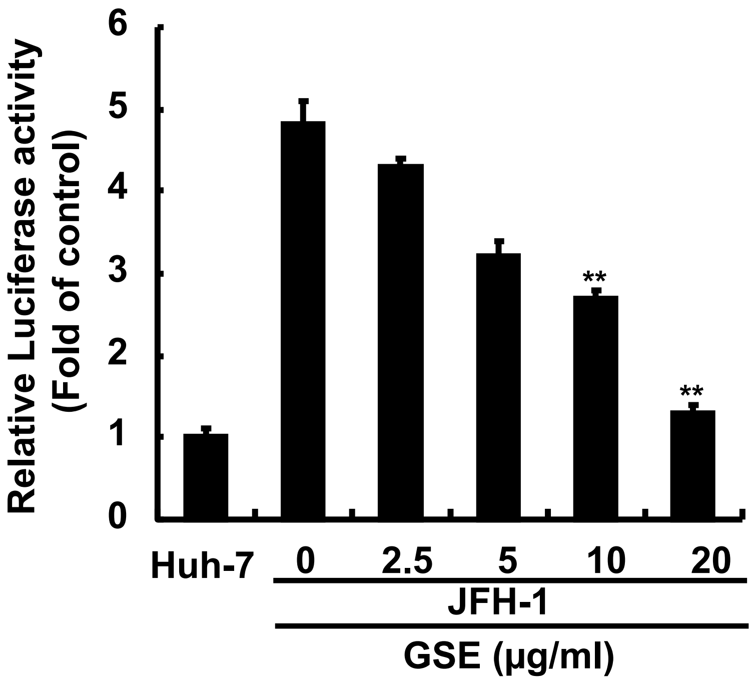

B

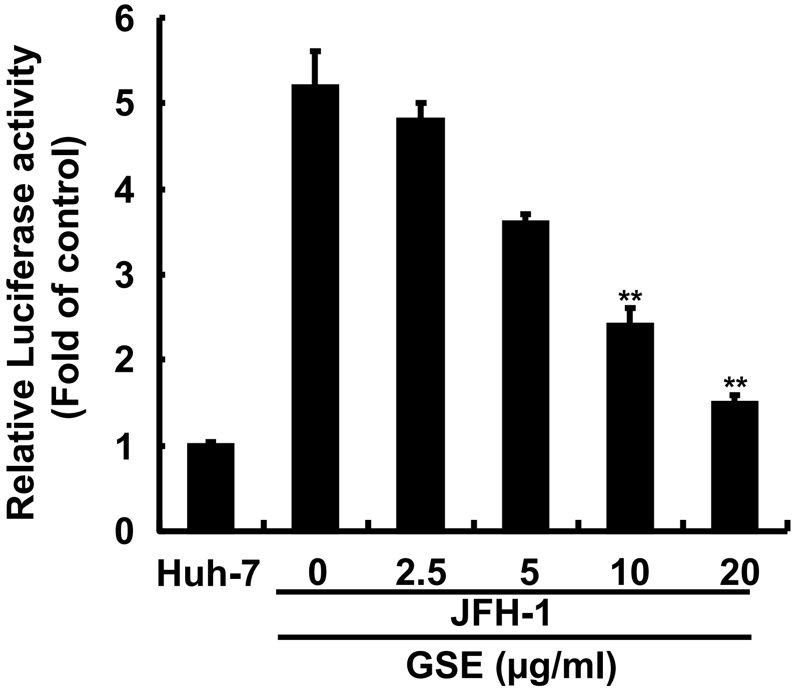

Supplementary Figure S2

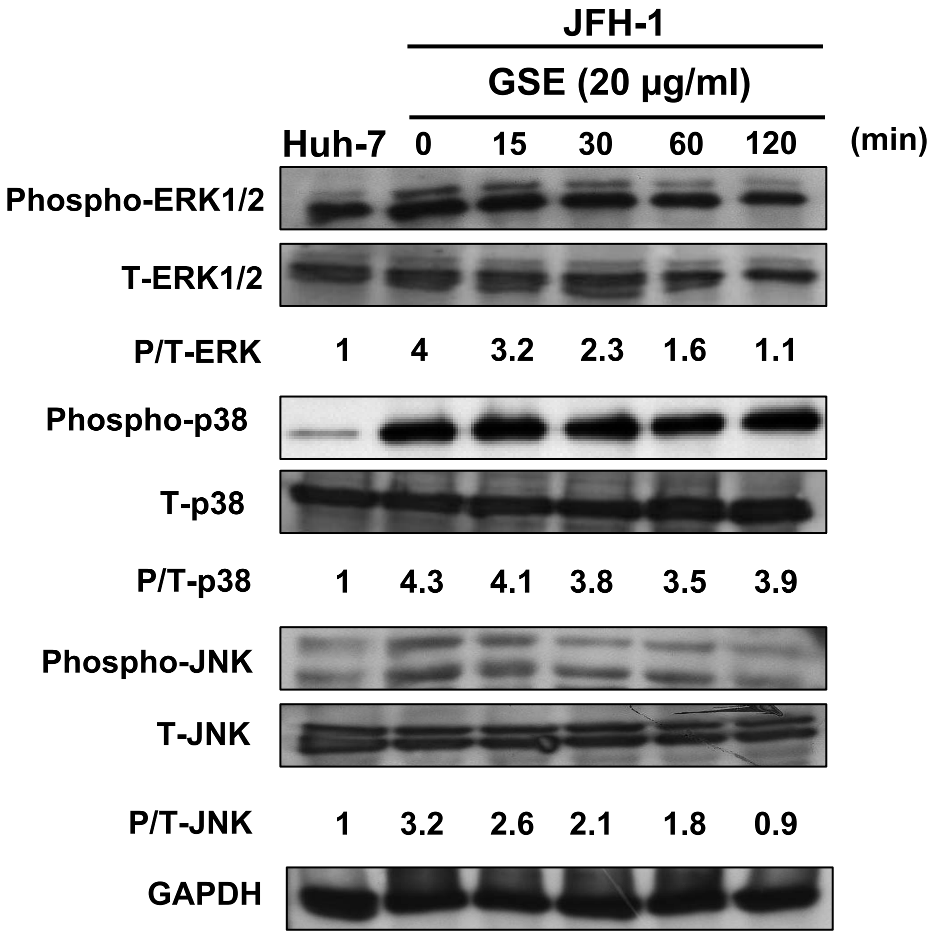

Supplement: Supplementary file 1 [file Presentation_1.PDF]
